# Exploration of Galectin Ligands Displayed on Gram-Negative Respiratory Bacterial Pathogens with Different Cell Surface Architectures

**DOI:** 10.3390/biom11040595

**Published:** 2021-04-18

**Authors:** María A. Campanero-Rhodes, Ioanna Kalograiaki, Begoña Euba, Enrique Llobet, Ana Ardá, Jesús Jiménez-Barbero, Junkal Garmendia, Dolores Solís

**Affiliations:** 1 Instituto de Química Física Rocasolano, CSIC, Serrano 119, 28006 Madrid, Spain; ma.campanero@iqfr.csic.es (M.A.C.-R.); ikalograiaki.csic@gmail.com (I.K.); 2 CIBER de Enfermedades Respiratorias (CIBERES), Avda Monforte de Lemos 3-5, 28029 Madrid, Spain; ellobet@optimapharm.eu (E.L.); juncal.garmendia@unavarra.es (J.G.); 3 Instituto de Agrobiotecnología, CSIC-Gobierno Navarra, Avda Pamplona 123, 31192 Mutilva, Spain; beuba@alumni.unav.es; 4 Optimapharm, ParcBit Edifici Disset A2, 07121 Palma, Spain; 5 CIC bioGUNE, Basque Research Technology Alliance, BRTA, Bizkaia Technology Park, Building 800, 48160 Derio, Spain; aarda@cicbiogune.es (A.A.); jjbarbero@cicbiogune.es (J.J.-B.); 6 Ikerbasque, Basque Foundation for Science, 48009 Bilbao, Spain; 7 Department Organic Chemistry II, Faculty of Science and Technology, UPV-EHU, 48940 Leioa, Spain

**Keywords:** bacteria microarrays, galectins, host–pathogen interactions, *Klebsiella pneumoniae*, non-typeable *Haemophilus influenzae*, lipopolysaccharides, lipooligosaccharides

## Abstract

Galectins bind various pathogens through recognition of distinct carbohydrate structures. In this work, we examined the binding of four human galectins to the Gram-negative bacteria *Klebsiella pneumoniae* (Kpn) and non-typeable *Haemophilus influenzae* (NTHi), which display different surface glycans. In particular, Kpn cells are covered by a polysaccharide capsule and display an O-chain-containing lipopolysaccharide (LPS), whereas NTHi is not capsulated and its LPS, termed lipooligosacccharide (LOS), does not contain O-chain. Binding assays to microarray-printed bacteria revealed that galectins-3, -4, and -8, but not galectin-1, bind to Kpn and NTHi cells, and confocal microscopy attested binding to bacterial cells in suspension. The three galectins bound to array-printed Kpn LPS. Moreover, analysis of galectin binding to mutant Kpn cells evidenced that the O-chain is the docking point for galectins on wild type Kpn. Galectins-3, -4, and -8 also bound the NTHi LOS. Microarray-assisted comparison of the binding to full-length and truncated LOSs, as well as to wild type and mutant cells, supported LOS involvement in galectin binding to NTHi. However, deletion of the entire LOS oligosaccharide chain actually increased binding to NTHi cells, indicating the availability of other ligands on the bacterial surface, as similarly inferred for Kpn cells devoid of both O-chain and capsule. Altogether, the results illustrate galectins’ versatility for recognizing different bacterial structures, and point out the occurrence of so far overlooked galectin ligands on bacterial surfaces.

## 1. Introduction

The innate immune system is the first line of defense against invading pathogens. Besides general physical, chemical, and cellular mechanisms, a variety of receptors of the innate immune system recognize different structures on pathogens’ surfaces for triggering specific responses, including lectins that target microbial glycans. Many of such lectins are presented on the surface of phagocytic cells while others are secreted, as, e.g., the galectins. While several innate immune lectins primarily recognize non-self carbohydrate patterns, galectins can recognize host-like structures that are displayed by several pathogens to masquerade as “self” and, thereby, avoid, subvert, or inhibit host immune responses, a mechanism known as molecular mimicry [1].

Galectins share a carbohydrate-recognition domain (CRD) with β-sandwich fold and galactoside-binding ability, although the fine glycan-binding specificity of different galectins may differ significantly [2]. Based on their structural organization, galectins are classified into three subgroups, i.e., (i) proto type galectins, composed of one or two identical CRDs that form non-covalent homodimers; (ii) chimera type, composed of one CRD linked to a non-lectin N-terminal region; and iii) tandem-repeat type, which contain two different CRDs covalently connected by a linker peptide. Galectins are involved in many different biological phenomena, including inflammation and immunity [3,4]. During infection, human galectins have been shown to exert various immunoregulatory roles, as recruitment of immune cells to the site of infection, promotion of neutrophil function, or stimulation of the bactericidal activity of infected macrophages [5,6,7,8]. In addition, selective galectin binding to a diversity of pathogens, including viruses, Gram-negative and -positive bacteria, fungi, and parasites, has been observed, with galectin-, pathogen-, and host context-specific consequences [7,8,9,10]. The particular ligands recognized by galectins on these various pathogens must be diverse, as their cell surface architectures and displayed glycans are different. 

A major surface component of Gram-negative bacteria is the lipopolysaccharide (LPS). The LPS is anchored into the outer membrane through a highly conserved lipid A moiety linked to a core oligosaccharide, which in turn is linked to an O-polysaccharide chain (also known as O-antigen), built with repeating saccharide units, that constitutes the outermost part of the LPS [11]. The precise composition and sequence of the O-chain may significantly differ among bacterial species and even between strains of a given bacterial species. Such diversity could lie behind the selective recognition of particular bacterial strains by certain galectins. As an example, human galectin-3 (Gal-3), the unique galectin of chimera type, as well as human tandem-repeat type galectins 4 (Gal-4) and 8 (Gal-8) recognize strains of *Klebsiella pneumoniae* displaying LPSs with O-chains containing blood group-like epitopes [12,13]. Moreover, binding of Gal-3 to the LPS isolated from *K. pneumoniae* (serotype O1) and other Gram-negative bacteria has been reported [14,15].

Interestingly, Gal-8 has also been found to recognize different strains of nontypeable (non-capsulated) *Haemophilus influenzae* (NTHi) [16], although the LPS of this bacterium does not present O-chain and is therefore referred to as lipooligosaccharide (LOS). In particular, the LOS of NTHi comprises a conserved heptose (Hep) trisaccharide inner core, linked to lipid A by a single 2-keto-3-deoxyoctulosonic acid (Kdo) residue [17]. Each Hep of the inner core can be a point for addition of strain-specific and phase-variable short carbohydrate extensions that constitute the outer core, which may also mimic host glycan epitopes and could potentially serve as galectin ligand. Other carbohydrate structures on the bacterial surface might also be recognized, as was inferred in a previous study on the binding of two model galactose-specific lectins to NTHi entire cells and isolated LOSs [18].

Prompted by these observations, in this work we comparatively examined the binding of four human galectins belonging to the three structural subgroups, i.e., proto-type galectin-1 (Gal-1), chimera type Gal-3, and tandem-repeat type Gal-4 and Gal-8, to *K. pneumoniae* and NTHi entire cells and isolated LPS/LOSs. Taking advantage of the availability of isogenic mutants lacking key carbohydrate structures, *K. pneumoniae* O1:K2 strain 52145 (hereafter referred to as Kpn), one of the most clinically prevalent serotypes, and NTHi strain 375 (hereafter referred to as NTHi), previously found to be recognized by Gal-8 [16], were selected for this study. Microarray binding assays, assisted by confocal microscopy, revealed galectin-selective recognition of wild type and mutant bacterial cells and LPS/LOSs. The results evidenced the versatility of galectins in the recognition of bacteria displaying different carbohydrate epitopes and cell surface architectures.

## 2. Materials and Methods

### 2.1. Bacteria Culture and Labelling and LPS/LOSs Isolation

*K. pneumoniae* strains used in this study included strain 52145 (wild type) and its mutants 52O21, deficient in the LPS O-chain (here designated Kpn OPS-), Δ*wca*_K2_, deficient in the polysaccharide capsule (designated Kpn CPS-), and Δ*waaL*Δ*wca*_K2_, deficient in both O-chain and capsule (designated Kpn OPS- CPS-) (see [19] for a detailed description of wild type and mutant strains). NTHi strains used included NTHi375 (wild type) and its mutants Δ*lgtF* (which lacks the LOS extension at the proximal Hep residue), Δ*lpsA* (which lacks the LOS extension at the distal Hep residue), Δ*opsX* (which lacks all outer and inner core sugars of the LOS), and Δ*ompP5*, a mutant lacking the major outer membrane protein P5 and found to produce higher amounts of LOS with identical structure to that of the wild type strain [18]. Bacterial cells were grown, fixed with paraformaldehyde, and fluorescently labelled with SYTO-13 (Invitrogen) as previously described [16,19]. Labelling efficiency was assessed by measuring the fluorescence intensity of bacteria suspensions at OD_600_ = 1 in 10 mM Tris/HCl, pH 7.8, 0.15 M NaCl, using a Horiba Jobin Yvon Fluoromax-4 spectrofluorometer.

LPSs were purified from 10 mg of lyophilized bacterial cells using the Tri-Reagent extraction method, as previously described [20]. To avoid co-purification of capsular polysaccharides, Kpn LPS was purified from the CPS- mutant Δ*wca_K2_*. LPSs were further treated with DNase, RNase and proteinase K before precipitation with 0.375 M magnesium chloride in 95% ethanol. NTHi wild type, Δ*lgtF*, and Δ*lpsA* LOSs were isolated from the aqueous layer of phenol-treated bacteria suspensions by precipitation with methanol containing 1% sodium acetate-saturated methanol [18]. For practical reasons, the wild type LOS was isolated from the Δ*ompP5* mutant.

### 2.2. Galectins

Human Gal-1 (unlabelled) and *N*-terminally His-tagged human Gal-3, Gal-4, and Gal-8 were obtained from ATGen Ltd. A second preparation of recombinant human Gal-1 was produced in BL21 *E. coli* cells via induction with isopropyl β-D-1-thio-galactopyranoside [21]. Following cell lysis, Gal-1 was purified by affinity chromatography on lactose-agarose (Sigma-Aldrich), using lactose-containing buffer as eluant. After extensive dialysis of the eluted fraction, Gal-1 purity was checked by SDS-PAGE and LC-MS, and the absence of lactose was confirmed by NMR spectroscopy [21].

### 2.3. Microarray Binding Assays 

Bacterial cells, purified LPS/LOSs, and control glycoproteins were printed on either single-pad or 16-pad nitrocellulose-coated glass slides (Maine mfg FAST-slides or Grace Biolabs ONCYTE NOVA) using a manual glass-slide arraying system (V&P Scientific) or a non-contact arrayer (Sprint, Arrayjet Ltd.), respectively, essentially as described [19,22]. Probes were printed as triplicates in a dose–response format. Cy3 dye (GE Healthcare) was included in LPS and control glycoprotein solutions to enable post-array monitoring of the spots [23]. The microarrays were scanned with a GenePix 200-AL scanner (Axon, Molecular Devices) for SYTO-13 and Cy3 signals, using excitation wavelengths of 488 nm and 532 nm, respectively, and stored in a dry dark place until they were used. Fluorescence signals were quantified with the GenePix Pro 6.0 software (Molecular Devices).

For binding assays, the microarrays were first blocked for 1 h with 0.25% (*v*/*v*) Tween-20 in 5 mM sodium phosphate, pH 7.2, 0.2 M NaCl (PBS), then rinsed with PBS and overlaid for 2 h with a 20 μg/mL solution of the different galectins in PBS containing 0.1% (*v*/*v*) Tween-20 and 1 mM DTT (overlay buffer), in the absence or presence of 75 mM lactose plus 1 mg/mL asialofetuin (Sigma). After 4 washes with PBS, the slides were overlaid for 1 h either with biotin-labelled anti-human galectin 1 (Peprotech), for unlabelled Gal-1, or with a pre-complexed mixture of mouse anti-poly-His antibody and biotinylated anti-mouse IgG (Sigma) at 1 and 3 μg/mL, respectively, for His-tagged galectins. Binding was detected by incubating with AF647-labelled streptavidin (Invitrogen) at 1 μg/mL in overlay buffer, for 35 min. All the steps were carried out protected from light and at 20 °C. Finally, the slides were first washed thoroughly with PBS and then with water, and scanned using a GenePix 200-AL scanner (Axon, Molecular Devices). 

### 2.4. Confocal Microscopy

Suspensions of SYTO-13-labelled bacterial cells at OD_600_ = 0.5 in PBS 1 mM DTT were incubated for 60 min in the absence or presence of galectins at 12 μg/mL final concentration. Following incubation, bacteria suspensions were centrifuged, washed once with PBS 1 mM DTT, and then incubated for 45 min with biotinylated anti-human Gal-1 or with mouse anti-His antibody precomplexed with biotinylated anti-mouse antibody, as used for the microarray binding assays. After washing, bacteria were incubated with 1 μg/mL streptavidin–AlexaFluor-647 for 30 min, washed, and examined by confocal microscopy. All the steps were carried out protected from light and at 20 °C.

Images were collected with a Leica TCS SP5 AOBS or TCS SP8 STED 3× confocal microscope (Mannheim, Germany) with 63× oil immersion optics. Laser lines at 488 nm and 633 nm for excitation of SYTO-13 and AlexaFluor-647 were provided by an Ar laser and a DPSS laser or with a White Light Laser Leica, depending on the microscope used. Detection ranges were set to eliminate crosstalk between fluorophores.

## 3. Results

### 3.1. Microarray and Confocal Microscopy Analyses of Galectin Binding to Bacterial Cells

*K. pneumoniae* (Kpn) and nontypeable *H. influenzae* (NTHi) are two Gram-negative bacteria that display clearly distinguishable cell surface glycan structures. First, the LPS of Kpn cells contains an O-polysaccharide chain built with repeating disaccharide units, while NTHi cells display an O-chain-lacking LOS. Second, Kpn cells are coated by a polysaccharide capsule while NTHi cells are not capsulated. In this work, the impact of this different cell surface architecture on the recognition by galectins was evaluated by examining the binding to microarray-printed Kpn and NTHi cells of four galectins representative of the three structural subgroups, i.e., Gal-1, Gal-3, Gal-4, and Gal-8. This approach has proved to be efficient for detecting recognition of these bacteria by antibodies and receptors of the innate immune system [16,19].

**Figure 1:**
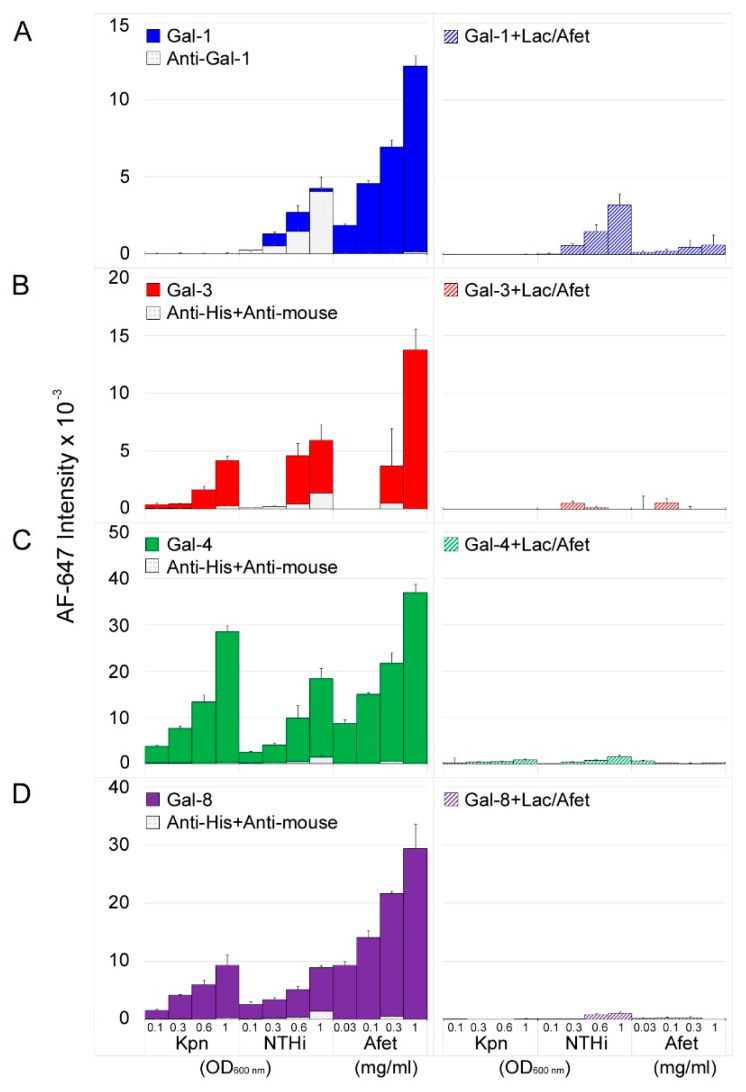
Galectin binding to Kpn and NTHi cells. Wild type bacterial cells were printed as triplicates at OD_600_ of 0.1, 0.3, 0.6, and 1 onto nitrocellulose-coated single-pad glass slides, using a manual arraying system. The pan-galectin ligand asialofetuin (Afet) was similarly printed at 0.03, 0.1, 0.3, and 1 mg/mL. The binding of galectins in the absence (left panel, solid columns) or presence of 75 mM lactose plus 1 mg/mL asialofetuin in solution (right panel, dashed columns) was assessed by incubation with biotinylated anti-Gal-1 or with mouse anti-poly-His antibody pre-complexed with biotinylated anti-mouse IgG, followed by incubation with AF647-streptavidin as final step. Blank experiments with antibodies in the absence of galectins were run in parallel (left panels, light grey columns). Data shown correspond to the mean of three different determinations and error bars indicate the standard deviation of the mean. From top to bottom, Gal-1 (**A**), Gal-3 (**B**), Gal-4 (**C**), and Gal-8 (**D**).

Clear differences in the behavior of the four galectins were visible (Figure 1). In the case of Gal-1, small signals were exclusively observed for printed NTHi (Figure 1A, left panel). However, competition assays carried out in parallel in the presence of lactose plus asialofetuin (a pan-galectin ligand [24]) in solution (Figure 1A, right panel) revealed a reduction of only 24–48% in the fluorescence signals with respect to the signals observed in the absence of competitors. Moreover, blank experiments run in the absence of Gal-1 yielded comparable or even higher intensities (Figure 1A, left panel), unveiling direct binding of the anti-Gal-1 antibody used in the detection protocol (see the Materials and Methods section). Altogether the results were indicative of negligible carbohydrate-mediated binding of Gal-1 to NTHi. To confirm this observation, the binding behavior of an in-house recombinantly produced Gal-1 was also examined. In this case, Gal-1 was biotinylated and fluorescently labelled streptavidin was employed for detection, thereby avoiding the use of the anti-Gal-1 antibody. As expected, no binding of biotin-labelled Gal-1 to the array-printed bacteria was detected. Of note, robust dose-dependent and inhibitable binding to asialofetuin printed in the array was observed for both Gal-1 preparations and detection systems, thereby proving Gal-1 activity (Figure 1A). In striking contrast, significant binding of Gal-3 to both Kpn and NTHi cells was observed, and fluorescent signals were almost completely abolished in the presence of lactose and asialofetuin in solution (Figure 1B). Finally, robust dose-dependent and inhibitable binding to Kpn and NTHi was observed for Gal-4 and Gal-8 (Figure 1C,D).

**Figure 2:**
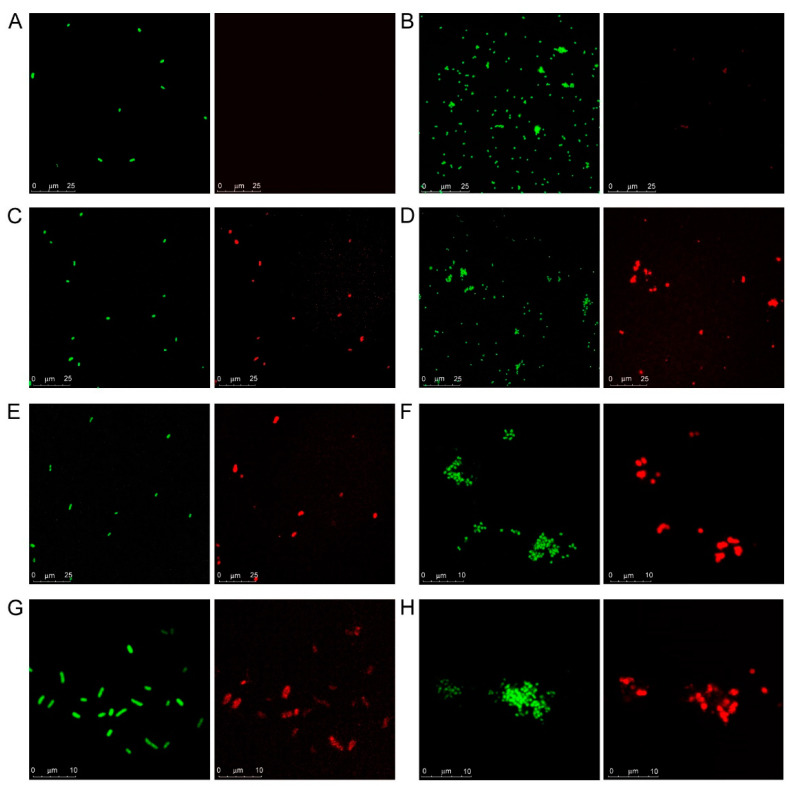
Confocal microscopy analysis of galectin binding to Kpn and NTHi cells. Suspensions of SYTO-13-labelled Kpn (panels **A**,**C**,**E**,**G**) and NTHi (panels **B**,**D**,**F**,**H**) cells at OD_600_ = 0.5 were incubated with 12 µg/mL of Gal-1 (**A**,**B**), Gal-3 (**C**,**D**), Gal-4 (**E**,**F**) or Gal-8 (**G**,**H**) and bound galectins were detected using the same protocol used in the microarray-binding assays, with incubation with AF647-streptavidin as final step. Green: SYTO-13 signals. Red: AF647 signals. For each image, scale bars are shown.

Confocal microscopy visualization of galectin binding to bacterial cells in suspension was fully in line with the results of the microarray assays. SYTO-13-stained bacteria were visible in the green channel while bound galectins were detected in the red channel (Figure 2), thus allowing spotting co-localization, indicative of galectin‒bacterial cell interaction. No red signals were detected for Kpn cells incubated with Gal-1 (Figure 2A), confirming that this galectin does not recognize the glycans displayed by the particular strain (Kpn52145) used in this study. For NTHi cells incubated with Gal-1, only very minor signals, predictably resulting from direct binding of the anti-Gal-1 antibody used in the detection protocol, were observed (Figure 2B). In contrast, binding of Gal-3, Gal-4, and Gal-8 to both Kpn and NTHi cells was visible (Figure 2C–H), supporting the results of the microarray assays. Interestingly, some bacterium- and galectin-specific differences were noticed. Thus, binding to Kpn cells was rather homogeneous for the three galectins (Figure 2C,E,G). However, selective binding to certain NTHi cells seemed to occur (Figure 2D,F,H), especially in the case of Gal-4 and Gal-8.

Having confirmed binding of Gal-3, Gal-4, and Gal-8 to Kpn and NTHi cells, the potential involvement of the LPS/LOS in the binding was next evaluated using selected mutants with altered LPS/LOS exposure and/or structure. 

### 3.2. Involvement of Kpn LPS in Galectin Binding to Bacterial Cells

**Figure 3:**
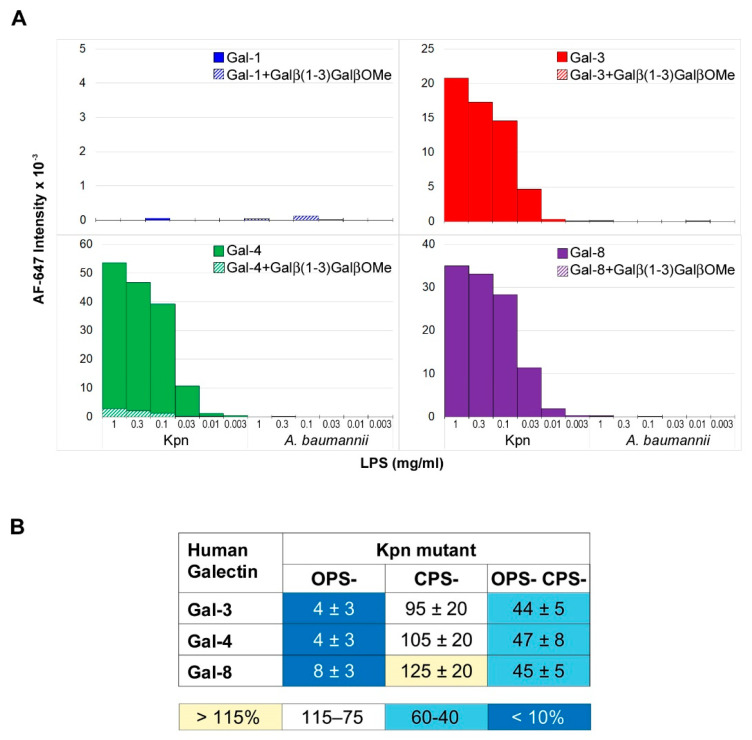
Microarray analysis of galectin binding to Kpn LPS and mutant cells. (**A**) The LPS from Kpn was printed as triplicates at six different concentrations (from 1 to 0.003 mg/mL), and the binding of galectins was assayed in the absence (solid columns) or presence of 10 mM Galβ(1–3)GalβOMe (dashed columns). The LPS from *A. baumannii* was also printed in the array as control of binding specificity. (**B**) Relative binding strength of galectins to Kpn mutant cells. The binding of galectins to array-printed wild type and mutant Kpn cells was assessed simultaneously, and the relative binding strength was calculated as percentage taking for each galectin the binding to wild type Kpn as 100%. Data shown correspond to mean values calculated from binding intensities to triplicate samples of bacteria printed at OD_600_ of 0.3, 0.6, and 1. Standard deviations of the mean are given.

As mentioned above, Gal-3 is known to interact with the LPS from *K. pneumoniae* (serotype O1) [14]. Therefore, this structure could serve as ligand on Kpn cells for this and other galectins. To explore this possibility, binding of Gal-1, Gal-3, Gal-4, and Gal-8 to the LPS isolated from the Kpn strain used in this work was first examined (Figure 3A). To this aim, the LPS was printed onto microarray slides and galectin binding was evaluated using the same detection protocols employed for binding assays to whole bacterial cells. Significant binding signals for Gal-3 were detected (Figure 3A), in line with previous observations. Moreover, robust binding of Gal-4 and Gal-8 was observed, whereas no binding of Gal-1 was detected.

**Figure 4:**
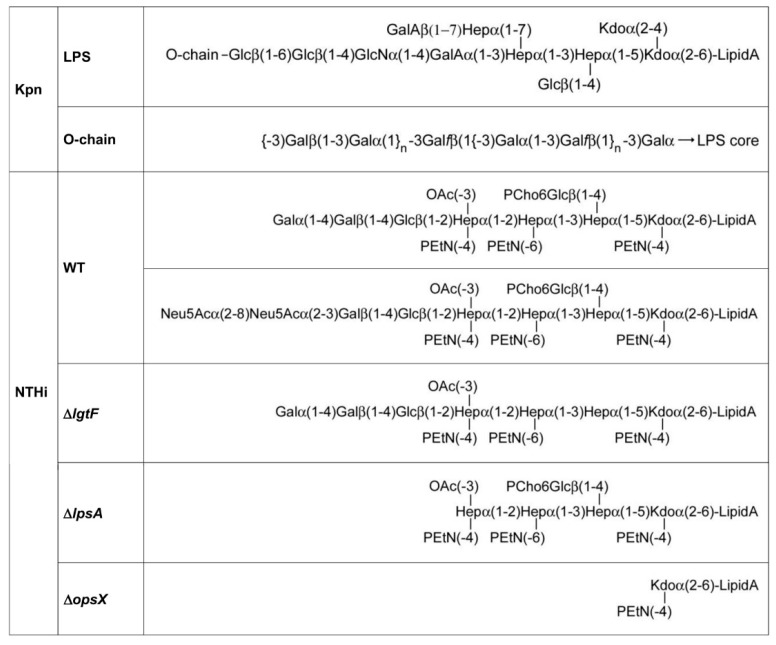
Structure of Kpn and NTHi LPS/LOSs. The structures shown correspond to *K. pneumoniae* 52145 and to NTHi 375 wild type and mutant strains. The two LOS glycoforms found for NTHi 375 [17] are shown. For the sake of simplicity, only the major, Gal-terminated glycoform is shown for the Δ*lgtF* mutant.

Additionally, important, none of the galectins bound to the LPS isolated from *Acinetobacter baumannii* (ATCC 19606), also printed in the array, supporting galectin-selective recognition of Kpn LPS. The O-chain of this LPS is built by repeating Galβ(1–3)Gal disaccharide units [25] (Figure 4), which could serve as a docking point for galectins. To evaluate this hypothesis, the inhibitory potential of the Galβ(1–3)GalβOMe disaccharide on the binding of Gal-3, Gal-4, and Gal-8 to array-printed Kpn LPS was examined. Indeed, galectin binding was almost completely inhibited in the presence of Galβ(1–3)GalOMe in solution (Figure 3A), substantiating the idea that Kpn LPS-binding galectins recognize the Galβ(1–3)Gal epitope present in the O-chain.

Based on the results of the LPS binding assays, three different Kpn mutants deficient in the LPS O-chain (OPS-), the polysaccharide capsule (CPS-), or both structures (OPS- CPS-) were selected for evaluating the contribution of LPS recognition to galectin binding to Kpn cells. Compared to wild type Kpn, binding signals of Gal-3, Gal-4, and Gal-8 to Kpn OPS- were drastically reduced, while similar or slightly more intense signals were observed for Kpn CPS- (Figure 3B). It has been reported that the O-chain in Kpn strains of serotype O1:K2 (as the strain used in this study) is accessible to antibody recognition despite the presence of the capsular polysaccharide [26]. Thus, the results obtained for Kpn OPS- and Kpn CPS- are compatible with the O-chain serving as galectin ligand on the surface of wild type Kpn cells. Interestingly, significant binding to the Kpn OPS- CPS- double mutant was observed for the three galectins. It is worth mentioning that no binding of Gal-1 to this mutant was detected. These results indicate that upon simultaneous deletion of these two major carbohydrate structures, i.e., the capsule and the LPS O-chain, other ligands for Gal-3, Gal-4, and Gal-8 become accessible for recognition on the bacterial surface. 

### 3.3. Involvement of NTHi LOS in Galectin Binding to Bacterial Cells

**Figure 5:**
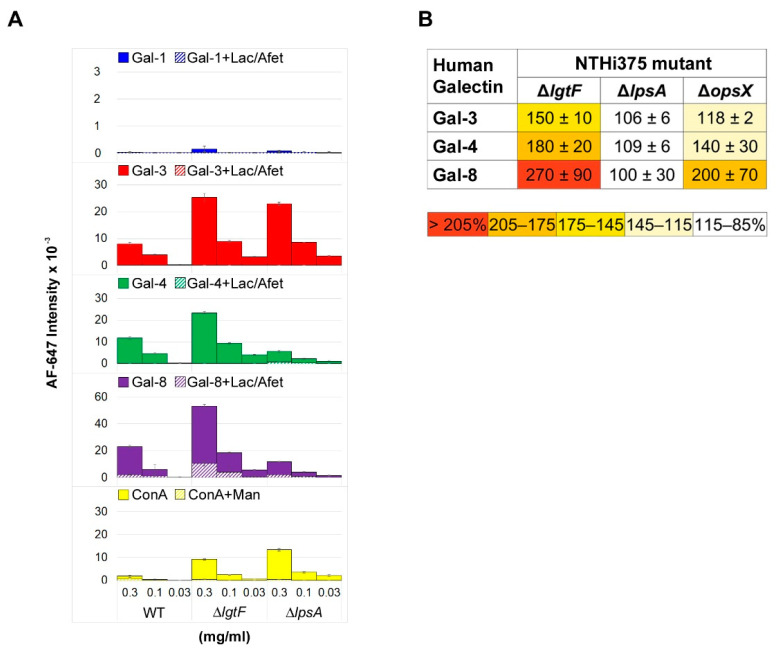
Microarray analysis of galectin binding to NTHi LOSs and mutant cells. (**A**) NTHi wild type, Δ*lgtF*, and Δ*lpsA* LOSs were printed as triplicates at three different concentrations (0.3, 0.1, and 0.03 mg/mL), and the binding of galectins was assayed in the absence (solid columns) or presence (dashed columns) of 75 mM lactose plus 1 mg/mL asialofetuin in solution, as described in Materials and Methods. Binding of biotin-labelled concanavalin A (ConA) at 20 µg/mL in the absence or presence of 0.1 M mannose, also detected by incubation with 1 µg/mL AF647-streptavidin, is also shown. (**B**) Relative binding strength of galectins to NTHi mutant cells. The binding of galectins to array-printed wild type and mutant NTHi cells was assessed simultaneously, and the relative binding strength was calculated as percentage taking for each galectin binding to wild type NTHi as 100%. Data shown correspond to mean values calculated from binding intensities to triplicate samples of bacteria printed at OD_600_ of 0.3, 0.6, and 1. Standard deviations of the mean are given.

In contrast to Kpn, NTHi does not present an O-chain-containing LPS. Therefore, the question arises whether the NTHi LOS could serve as ligand for galectins on the bacterial surface. To answer this question, the binding of Gal-1, Gal-3, Gal-4, and Gal-8 to the NTHi LOS was first examined. Three different LOSs were used for comparative analysis (Figure 4): (i) wild type LOS, (ii) LOS isolated from the NTHi mutant Δ*lgtF*, which lacks the extension at the proximal heptomannose (Hep I) residue, and (iii) LOS isolated from the NTHi mutant Δ*lpsA*, which lacks the galactose-containing extension at the distal heptomannose residue (Hep III). The three LOSs were printed onto microarray slides and galectin binding was examined in the absence or presence of lactose plus asialofetuin (Figure 5A).

Not surprisingly, no binding of Gal-1 to any of the array-printed LOSs was detected (Figure 5A). However, meaningful dose-dependent and inhibitable binding signals were observed for Gal-3, Gal-4, and Gal-8. LOS truncation at Hep I in NTHiΔ*lgtF* resulted in increased binding signals for the three galectins. This behavior was previously observed for the galactose-specific lectin from *Viscum album* and tentatively explained by an increase in accessibility to the distal galactose-containing extension in the absence of the Hep I branch [18]. On the other hand, LOS truncation at Hep III in the Δ*lpsA* mutant had different consequences depending on the galectin. For Gal-3, binding to NTHiΔ*lpsA* LOS was comparable to that to the NTHiΔ*lgtF* LOS (Figure 5A). Gal-3 has been reported to bind a range of “non-lactose” glycans, including mannose-based structures [27,28]. It is tempting to speculate that, in the absence of the LOS branch at Hep III, other glycan epitopes becoming exposed could serve as ligands for Gal-3. Indeed, binding assays with the mannose/glucose-specific lectin concanavalin A to the array-printed LOSs, carried out in parallel (Figure 5A, bottom panel), revealed a significant increase in the binding to the NTHiΔ*lpsA* LOS compared to wild type LOS, plausibly due to a greater exposure of sugar epitopes recognized by this lectin. In contrast, binding signals of Gal-4 and Gal-8 to the Δ*lpsA* LOS were notably smaller, down to levels of around 50% of the binding to wild type LOS (Figure 5A). These results were indicative of recognition of the galactose-containing Hep III branch by these two galectins. Thus, binding of Gal-3, Gal-4, and Gal-8 to the NTHi LOS was differentially affected by LOS truncation.

To evaluate whether LOS recognition correlates with binding to entire bacterial cells, binding assays to microarray-printed wild type and mutant NTHi cells were next performed. In addition to the NTHiΔ*lgtF* and NTHiΔ*lpsA* mutants, a strain lacking all outer and inner core sugars (Δ*opsX*, Figure 4, see also [16]) was included in the analysis. Starting with NTHiΔ*lgtF* cells, a noticeable increase in binding signals for the three galectins in comparison to wild type NTHi was observed. This correlation with LOS recognition points to the LOS serving as ligand on the bacterial surface. On the other hand, deletion of the galactose-containing branch at Hep III in the NTHiΔ*lpsA* mutant apparently had no detrimental consequences on galectin binding to entire cells. Moreover, complete deletion of the outer and inner core sugars in the NTHiΔ*opsX* mutant resulted in increased binding of the three galectins compared to wild type NTHi, albeit at different levels. Of note, no significant carbohydrate-mediated binding of Gal-1 to this mutant was detected. Thus, as observed for the Kpn OPS- CPS- double mutant, the results indicate that deletion of the oligosaccharide chain apparently favors binding of Gal-3, Gal-4, and Gal-8 to other ligands available on the bacterial surface.

## 4. Discussion

Galectins were originally defined as a family of animal lectins with β-galactoside-binding activity [29]. The fine oligosaccharide-binding specificity of galectins, however, may differ substantially, and a diversity of ligands have been found for different members of this family, even including mannose-based structures. Galectin-selective binding to many pathogens, ranging from viruses to parasites, has been reported. In this work, the recognition of two important respiratory pathogens included in the WHO priority pathogens list [30], Kpn and NTHi, by human Gal-1, Gal-3, Gal-4, and Gal-8 was examined. Gal-1 set itself apart from the other three galectins because it did not bind to any of these two Gram-negative bacteria. Failure of Gal-1 to recognize blood group B-positive *Escherichia coli* was also observed previously [12]. However, this galectin is known to bind to a variety of pathogens, including Nipah virus, HIV-1, influenza virus, and human T cell leukemia virus type 1 (HTLV-1) [31,32,33,34], as well as the Gram-positive bacterium *Streptococcus pneumoniae* and the parasite *Trichomonas vaginalis* [35,36], via recognition of surface carbohydrate structures, such as N-glycans from viral envelope glycoproteins, pneumococcal capsular polysaccharides, or the parasite lipophosphoglycan, respectively. In contrast, Gal-3, Gal,4, and Gal-8 did bind to Kpn and NTHi. Thus, Gal-1 selectivity in pathogen recognition manifestly differs from that of these three galectins.

Gal-3, Gal,4, and Gal-8 exhibited the same behavior when binding to Kpn. The three galectins use the O-chain of Kpn LPS as docking point on the bacterial surface, and binding to Kpn cells seems to be rather homogeneous, as visualized by confocal microscopy. However, some differences were discerned in the binding to NTHi. The three galectins were able to recognize the NTHi LOS, but the consequences of LOS truncation differed for Gal-3, for which deletion of the galactose-containing extension did not result in decreased binding. The unique ability of Gal-3 to bind a wide diversity of glycans, including, e.g., fungal β-1,2-linked mannans [27] and mycolic acids [37], or even non-carbohydrate structures, as found for gold porphyrin complexes [38], could lie beneath this behavior. Apparently, the LOS serves as ligand for the three galectins on the NTHi cell surface. However, the presence of other ligands seems evident, as deletion of the entire LOS oligosaccharide chain actually increased galectin binding, particularly for Gal-4 and Gal-8. A similar conclusion can be drawn for “nude” Kpn devoid of capsule and LPS O-chain, therefore resembling the surface architecture of NTHi. However, such ligands are definitely not accessible on the surface of wild type, i.e., capsulated, Kpn.

Recognition of the high molecular weight adhesin HMW1 by the galactose-specific agglutinin VAA was inferred in a previous study in which the binding of this lectin to NTHi strains expressing HMW1 or not was comparatively examined [18]. HMW1 is glycosylated at multiple sites with N-linked Gal/Gal-Glc units [39], and it is expressed by the particular NTHi strain studied here. Therefore, this adhesin could also serve as a galectin ligand. Interestingly, it has been reported that Gal-1 binds to several glycoproteins of the non-capsulated LOS-bearing bacterium *Chlamydia trachomatis* [40]. On the other hand, binding of the galactose-specific agglutinin from *Ricinus communis* to NTHi cells apparently involves recognition of sugar epitopes other than those displayed by the LOS and HMW1 [18]. These unidentified carbohydrate structures could also be potential ligands for galectins. Thus, any possible intra-strain heterogeneity regarding distribution, abundance and/or accessibility of these ligands on the NTHi surface could conceivably explain a selective binding of Gal-4 and Gal-8 to certain NTHi cells, as pointed out by the microscopy analysis. Overall, this study evidenced the versatility of galectins in the recognition of Gram-negative bacteria displaying different cell surface architectures, and draws attention to the occurrence of so far overlooked ligands for human galectins on bacterial surfaces.

Galectins’ plasticity in targeting diverse ligands in multifarious pathogens, including Gram-negative and -positive bacteria, virus, fungi, and parasites, points to a key role of these lectins in immune protection. Moreover, galectins are ubiquitously present in many metazoan phyla, ranging from vertebrates to sponges, and antimicrobial activities for galectins from phylogenetically very distant species, e.g., humans, shrimps, or oysters [12,13,41], have been described. Thus, this ancient family of lectins could serve as an evolutionarily conserved “Swiss army knife” of innate immunity.

## Data Availability

Data presented in this study are available on request from the corresponding author.
